# Genome sequence of *Haemophilus parasuis* strain 29755

**DOI:** 10.4056/sigs.2245029

**Published:** 2011-09-23

**Authors:** Michael A. Mullins, Karen B. Register, Darrell O. Bayles, David W. Dyer, Joanna S. Kuehn, Gregory J. Phillips

**Affiliations:** 1 Virus and Prion Research Unit, USDA/Agricultural Research Service/National Animal Disease Center, Ames, IA, USA; 2 Bacterial Diseases of Livestock Research Unit, USDA/Agricultural Research Service/National Animal Disease Center, Ames, IA, USA; 3 Department of Microbiology and Immunology, University of Oklahoma Health Sciences Center Biomedical Research Center, Oklahoma City, OK, USA; 4 Department of Veterinary Microbiology and Preventive Medicine, Iowa State University College of Veterinary Medicine, Ames, IA, USA

**Keywords:** *Haemophilus parasuis*, Glässer’s disease, swine

## Abstract

*Haemophilus parasuis* is a member of the family *Pasteurellaceae* and is the etiologic agent of Glässer’s disease in pigs, a systemic syndrome associated with only a subset of isolates. The genetic basis for virulence and systemic spread of particular *H. parasuis* isolates is currently unknown. Strain 29755 is an invasive isolate that has long been used in the study of Glässer’s disease. Accordingly, the genome sequence of strain 29755 is of considerable importance to investigators endeavoring to understand the molecular pathogenesis of *H. parasuis*. Here we describe the features of the 2,224,137 bp draft genome sequence of strain 29755 generated from 454-FLX pyrosequencing. These data comprise the first publicly available genome sequence for this bacterium.

## Introduction

*H. parasuis* is an obligate pathogen of swine [1]. The bacterium is often carried in the nasal passages [2], but not the lungs [3], of healthy pigs. Through unknown mechanisms some strains can spread systemically and may be isolated from the meninges, lungs, serosa, joints, and blood. *H. parasuis* strain 29755 (IA84-29755), though not the type strain, has been used extensively in a variety of investigations [4-8] and is the most fully characterized strain of the species. Originally cultured at Iowa State University from a pig exhibiting Glässer’s disease, 29755 is a serovar 5 isolate [9], a class recognized as highly virulent and frequently isolated from respiratory and systemic sites [9,10]. Of the 15 recognized serovars, serovar 5 strains are isolated more frequently worldwide than any other [11]. Strain 29755 has been used as a component of at least one commercially available *H. parasuis* vaccine (Suvaxyn M. hyo – parasuis, Fort Dodge Animal Health).

## Classification and features

**Figure 1:**
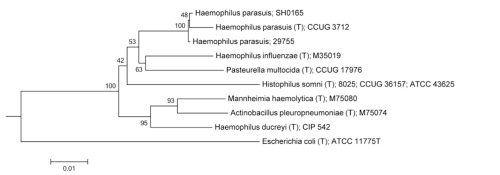
Phylogenetic tree based on 16S rRNA of *H. parasuis* 29755 and type strains of some closely related species and other genera within the *Pasteurellaceae*. Also included is the only additional *H. parasuis* strain for which a genome sequence has been reported, SH0165. The tree was generated with the tree-builder available from the Ribosomal Database Project[27] using the Weighbor (weighted neighbor-joining) algorithm [28] with Jukes-Cantor distance correction [29]. Numbers to the left of branches indicate the percentage of trees in which each branch was represented in 100 replicates. An *E. coli* type strain was used as an outgroup.

The genus *Haemophilus* belongs to the *Gammaproteobacteria* and is classified in the family *Pasteurellaceae* [12] (Table 1). A phylogenetic tree based on 16S ribosomal RNA sequences is depicted in Figure 1 for *H. parasuis* and related organisms.

**Table 1:** MIGS classification and general features of *H. parasuis* strain 29755.

**MIGS ID**	**Property**	**Term**	**Evidence code**
	Current classification	Domain *Bacteria*	TAS [13]
		Phylum *Proteobacteria*	TAS [14]
		Class *Gammaproteobacteria*	TAS [15,16]
		Order *Pasteurellales*	TAS [15,17]
		Family *Pasteurellaceae*	TAS [18,19]
		Genus *Haemophilus*	TAS [20-22]
		Species *Haemophilus parasuis*	TAS [20,23]
		Strain 29755	
		Serotype 5	
	Gram stain	negative	TAS [1]
	Cell shape	rods (pleomorphic)	TAS [1]
	Motility	nonmotile	TAS [1]
	Sporulation	non-sporulating	TAS [1]
	Temperature range	mesophile (20°C-37°C)	TAS [12]
	Optimum temperature	35°C-37°C	TAS [12]
	Carbon source	saccharolytic	TAS [24]
	Energy source	chemoorganotroph	TAS [24]
	Terminal electron receptor	Oxygen	TAS [25]
MIGS-6	Habitat	Host, swine upper respiratory tract	TAS [1]
MIGS-6.3	Salinity	1-1.5%	TAS [12]
MIGS-22	Oxygen requirement	facultative	TAS [12]
MIGS-15	Biotic relationship	obligate pathogen of swine	TAS [1]
MIGS-14	Pathogenicity	mild to severe	TAS [1]
MIGS-4	Geographic location	Iowa	NAS
MIGS-5	Sample collection time	1970s	NAS
MIGS-4.1	Latitude	not reported	
MIGS-4.2	Longitude	not reported	
MIGS-4.3	Depth	not reported	
MIGS-4.4	Altitude	not reported	

Evidence codes - NAS: Non-traceable Author Statement (i.e., not directly observed for the living, isolated sample, but based on a generally accepted property for the species, or anecdotal evidence). These evidence codes are from of the Gene Ontology project [26]

**Figure 2:**
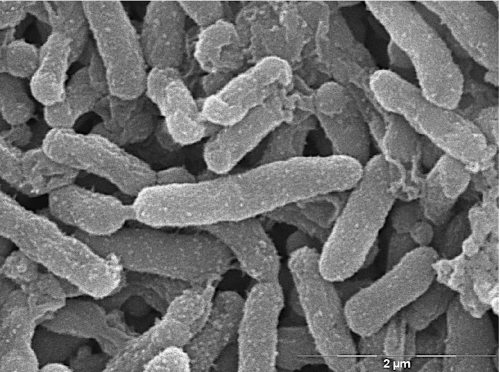
Scanning electron micrograph of *H. parasuis* 29755

*H. parasuis* is a small, non-motile, rod-shaped bacterium [1] (Figure 2). The presence of a capsule is variable and may affect colony and cellular morphology [30]. Growth of the bacterium *in vitro* is dependent on the coenzyme nicotinamide adenine dinucleotide (NAD, or V factor) [31] but, in contrast to some other members of the genus, does not require porphyrins like hemin (X factor) [32]. Plating on Casman Agar Base (BBL) supplemented with 1% (w/v) NAD (Sigma) and 5% GIBCO filtered horse serum (Invitrogen) or on chocolate agar produces small, translucent colonies that appear within 24 hours and reach full size in approximately two days. Colonies are nonhemolytic when grown on blood agar [1]. *H. parasuis* grows under normal atmosphere at 37°C, although added humidity and 5% CO_2_ may improve growth.

## Genome sequencing and annotation

### Genome project history

*H. parasuis* strain 29755 was selected for sequencing because it has long been used in the study of Glässer’s disease. Pyrosequencing (454 Life Sciences) was performed at the State University of New York, University at Buffalo Center of Excellence in Bioinformatics and Life Sciences. The draft genome sequence is deposited in GenBank (NZ_ABKM00000000). Summary project information is shown in Table 2 according to the Minimum Information about a Genomic Sequence (MIGS) recommendations [34] and the genome content is summarized in Table 3.

**Table 2:** Genome sequencing project information

**MIGS ID**	**Property**	**Term**
MIGS-28	Libraries used	one 454 pyrosequence standard library
MIGS-29	Sequencing platforms	454 (FLX)
MIGS-30	Assemblers	Newbler
MIGS-31	Finishing quality	draft
MIGS-31.2	Fold coverage	28×
MIGS-32	Gene calling method	Glimmer, GeneMark [33]
	Genome Database release	February 14, 2008
	Genbank ID	NZ_ABKM00000000
	Genbank Date of Release	February 14, 2008
	GOLD ID	-
	Project relevance	food animal pathogenesis

**Table 3:** Genome statistics

**Attribute**	**Value**	**% of total^a^**
Size (bp)	2,224,137	100.0%
G+C content (bp)	867,413	39.0%
Coding region (bp)	1,890,516	85.0%
Total genes	2,309	100.0%
RNA genes	58	2.5%
Protein-coding genes	2,244	97.2%
Pseudogenes	none^b^	0.0%
Genes in paralog clusters	nd^c^	-
Genes assigned to COGs	1,926	83.4%
PSORT cytoplasmic	1,181	50.4%
PSORT extracellular	5	0.2%
PSORT outer membrane	51	2.2%
PSORT periplasmic	52	2.2%
PSORT unknown	1,055	45.0%

^a^ Based either on the size of the genome in base pairs or the total number of protein coding genes in the annotated genome

^b^ Based on preliminary analysis the of draft genome

^c^ nd = not determined

### Growth conditions and DNA isolation

*H. parasuis* 29755 was grown from a frozen seed stock for two days under 5% CO_2_ at 37°C on Casman Agar Base (BBL) supplemented with 1% (w/v) NAD (Sigma) and 5% GIBCO filtered horse serum (Invitrogen). Following growth, a single colony was used to inoculate 5 ml of brain-heart infusion medium supplemented with 10 μg/ml NAD and 10 μg/ml hemin (sBHI) and the culture was incubated overnight at 37°C and 185 rpm. The next day, 2 ml of the culture were added to 100 ml of sBHI and the bacterium was again allowed to grow overnight to stationary phase at 37°C and 185 rpm. Bacterial cells were pelleted by centrifugation at 4000 × *g* for 10 minutes. The pellet was resuspended and used as the source of genomic DNA purified with the QIAGEN Blood & Cell Culture DNA Kit, as recommended by the manufacturer. The final preparation contained 1.12 μg/ul genomic DNA as determined by UV absorption spectrometry.

### Genome sequencing and assembly

Library preparation yielded 9.65 × 10^8^ molecules/μl of DNA with a mean size of approximately 600 nucleotides, as determined with a RNA6000 Pico chip on an Agilent 2100 Bioanalyzer. Emulsion PCR was performed at a concentration of 2 molecules per bead. Following sequencing, contigs were assembled using the 454 Newbler assembler.

### Genome annotation

Genes were identified manually using GeneMark and automatically using Glimmer as part of the NCBI draft genome submission pipeline. Translated protein sequences were analyzed using PSORTb v.2.0 [35] to predict final location within the cell and assigned to COG functional categories (Table 4).

**Table 4:** Number of genes associated with the general COG functional categories

**Code**	**Value**	**%age**^a^	**Description**
J	168	6.55	Translation
A	1	0.03	RNA processing and modification
K	127	4.96	Transcription
L	166	6.48	Replication, recombination and repair
B	0	0.00	Chromatin structure and dynamics
D	33	1.29	Cell cycle control, mitosis and meiosis
Y	0	0.00	Nuclear structure
V	32	1.25	Defense mechanisms
T	48	1.87	Signal transduction mechanisms
M	134	5.23	Cell wall/membrane biogenesis
N	16	0.62	Cell motility
Z	0	0.00	Cytoskeleton
W	24	0.94	Extracellular structures
U	75	2.93	Intracellular trafficking and secretion
O	101	3.94	Posttranslational modification, protein turnover, chaperones
C	115	4.49	Energy production and conversion
G	139	5.42	Carbohydrate transport and metabolism
E	175	6.83	Amino acid transport and metabolism
F	57	2.22	Nucleotide transport and metabolism
H	97	3.78	Coenzyme transport and metabolism
I	43	1.68	Lipid transport and metabolism
P	116	4.53	Inorganic ion transport and metabolism
Q	25	0.96	Secondary metabolites biosynthesis, transport and catabolism
R	234	9.13	General function prediction only
S	197	7.69	Function unknown
-	440	17.16	Not in COGs

^a^ Based on the total number of protein coding genes in the annotated genome

## Genome properties

**Figure 3:**
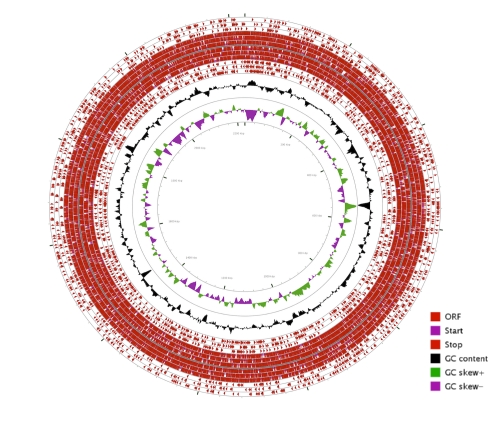
Graphical circular map of the *H. parasuis* 29755 draft pseudogenome. From the outside to the center: open reading frames (ORFs) on the forward strand (one ring for each reading frame), start and stop codons for forward and reverse strands, ORFs on the reverse strand, GC content, and GC skew. The map was generated using CGView Server [36,37].

The draft genome is 2,224,137 bp and is likely comprised of one circular chromosome with a G+C content of approximately 39% (Figure 3). For display, contigs were assembled end-to-end with twenty “N” bases between contigs. Orientation and order of contigs will change when the genome sequence is closed.
